# Optimization of electric bicycle for youths with disabilities

**DOI:** 10.1186/2193-1801-3-646

**Published:** 2014-11-01

**Authors:** Tobias Blumenstein, Hilar Zeitlmann, Ana Alves-Pinto, Varvara Turova, Renée Lampe

**Affiliations:** Markus Würth Stiftungsprofessur, Munich, Germany; Forschungseinheit für Cerebralparesen und Kinderneuroorthopädie der Buhl-Strohmaier Stiftung, Department of Orthopedics, Clinic ‘rechts der Isar’, Technical University of Munich, Ismaningerstr. 22, Munich, 81675 Germany

**Keywords:** Cerebral palsy, Cycling, Ultrasonic sensors, Acoustic feedback, Space orientation

## Abstract

Cerebral palsy is a group of neurodevelopmental disorders that affect a person’s ability to move and to maintain balance and posture. People with cerebral palsy have also perception and space orientation deficits so that special assistance devices should be developed to compensate these handicaps. The objective was to optimize an adapted electric bicycle (E-bike) for youths with neurodevelopmental disorders. An adapted E-bike was provided with ultrasonic sensors that measure distances to objects. If the distance to other objects reduces, an acoustic signal is sent. Additionally, a self-created force plate was fixed on the pedal to evaluate the muscle performances during biking. An experiment with the ultrasound warning system confirmed that acoustic feedback was helpful in avoiding obstacles. The measurement of the blood pressure, the heart frequency and the pedaling force during biking approved that the training condition of the test person can be registered and enables tuning the power of the electric motor to individual requirements. The results demonstrate that an adapted E-bike can be improved to provide better space orientation for people with perceptual disorders and to measure training conditions of patients. Moreover, these enable individual adjustment of the electric motor power to optimize comfort and therapy effect.

## Background

Special therapy programmes for children and youths with cerebral palsy enclose dynamic cycling on adapted bikes (see e.g. (Pickering et al. 2013)). Such training should improve the coordination, the balance, and also the physical condition (Williams and Pountney 2007; Siebert et al. 2010). In general, one distinguishes between active and passive cycling. In active cycling the bike pedals are stepped by the person himself/herself, whereas in passive cycling the legs follow the motion of the pedals (Bar-Haim et al. 2007). In both types of cycling, the therapy effect is due to reciprocal movement with alternating flexion and extension in the joints. This can help to strengthen the muscles (Lauer et al. 2008; Johnston and Wainwright 2011) and can prevent contractures of the joints (Rohde 2003).

Active cycling can improve integration, physical activity, and mobility of people with cerebral palsy. However, one should take into account that problems of perception and space orientation deficits lead to rarely use of bicycles in traffic. Therefore, one comes to the idea of extending the cycling therapy by an option that can compensate the decreased sense of orientation in space and even train the spatial perception. Our suggestion is to equip an E-bike with ultrasonic sensors that can measure distances to objects and give acoustic feedback if the distance becomes less than a defined threshold. Such a system will provide a patient with helpful information on his position with respect to environments and will make cycling more safety. Additionally, the force generated by the legs and the cadence during cycling can be evaluated by using a force measurement plate, which is integrated in the pedal. This will enable tuning the electric motor power to optimize the individual training load. Furthermore, it is useful to integrate a blinker to make the wish of changing the direction visible.

This study describes our experiences with the design and implementation of the above mentioned modifications and presents some experimental results.

## Material and methods

An E-bike with an adapted 250 W electric motor was used as a basic vehicle. The motor is controlled via a motion sensor that recognizes only the movement of the pedal.

**Figure 1:**
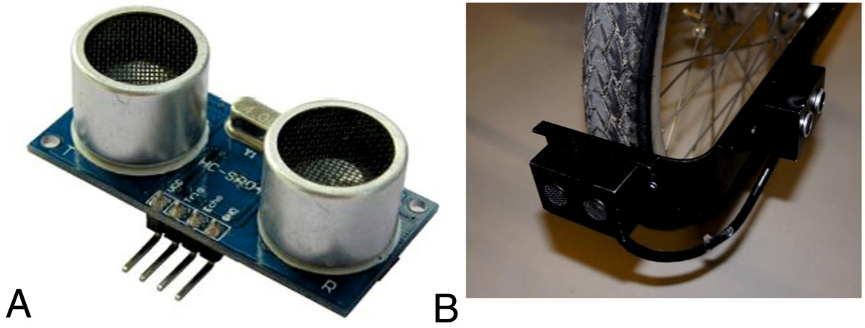
**Distance detection design. (A)** Ultrasonic sensor; **(B)** Retainer with mounted distance measurement unit.

To measure distances, the ultrasonic ranging module HC-SR04 (see Cytron Technologies 2013 and Figure 1A) with the ranging accuracy of 3 mm was used. With this sensor, distances between 2 cm and 400 cm can be registered. For mounting the distance measurement unit, an aluminum retainer (see Figure 1B) was used. It ensures that the sensors will always be in the same position related to the wheels. Four sensors were placed on the wheels of the bicycle, two in driving direction and one on each side. The position of the sensors allowed a regular assessment of the direction of travel.

The ultrasonic sensors are controlled by an Arduino Uno microcontroller. The microcontroller can be programmed with the special Arduino software. A corresponding programme code for the distance warning system was written.

To generate an acoustic signal, the piezo transducer KPM-G1205A-K6327 was used.

A motorbike LED-blinker DROP was utilized for indicating the changing of the direction. It was connected to a KEMO flasher board that enables the adjustment of the flash rate via a potentiometer.

For the power supply for the system, a nickel-metal hydride battery with an overall capacity of 2400 mAh was applied.

**Figure 2:**
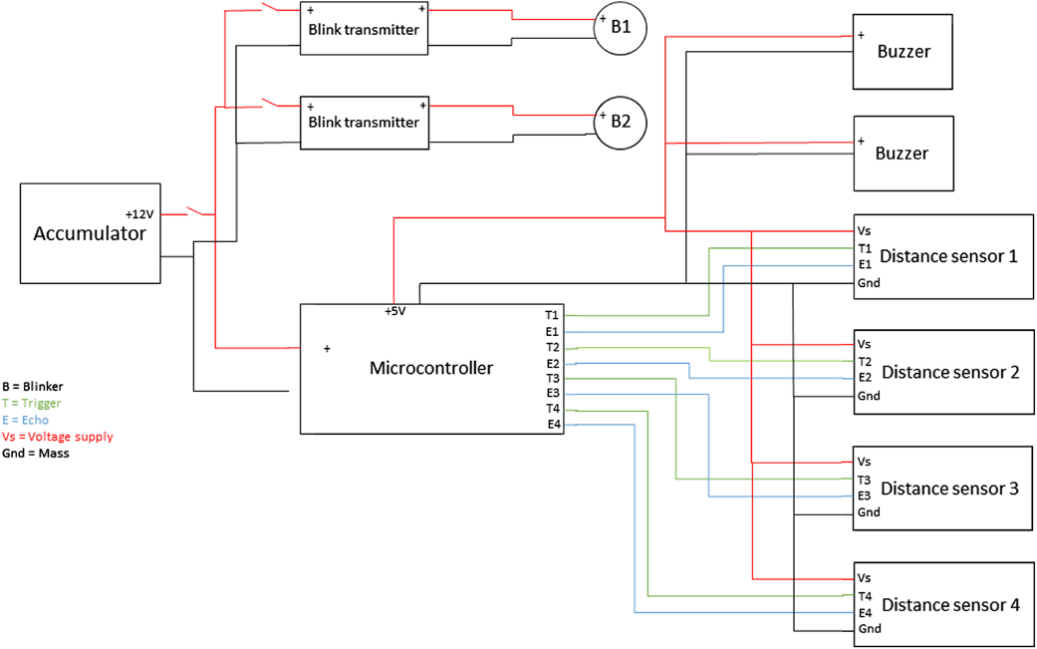
**Connection scheme of electric components.**

The connection scheme of electric components is presented in Figure 2. The system can be switched off with a toggle switch being integrated in the circuit. With this connector, the microcontroller board and the flasher are linked to the accumulator. The distance sensors and the buzzers are connected to the I/O ports and the 5 V power supply of the microcontroller.

**Figure 3:**
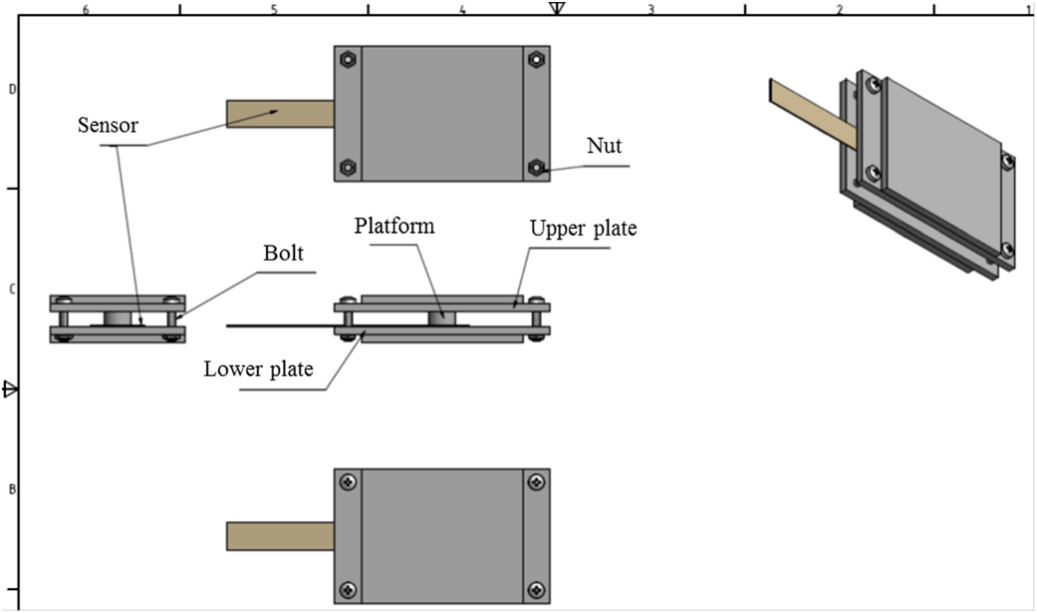
**Design of the force measurement plate.**

For the force measurement plate, a FlexiForce Sensor (Tekscan) with a sensing area of 10-mm diameter was used. The sensor was fixed between two aluminum plates (see Figure 3 for the corresponding CAD-drawing). The upper plate was supplied with a special platform to ensure that the force is only applied to the active region of the sensor. After relieving the load the plates should return to the initial position. To this end, four springs were fixed on the borders of the lower plate. The force sensor was calibrated using known weights.

The bike computer ROX 5.0 (Sigma) was used to register pedaling rate and velocity. The measured data are sent from the sensors to the bike computer over a wireless communication channel. The evaluation of the recorded data can be performed on a pc by connecting the bike computer to it.

Since the aim of the project was to test the functionality of the system, only one healthy subject was tested. In a following study the bicycle will be tested on several patients with disability.

## Results

**Figure 4:**
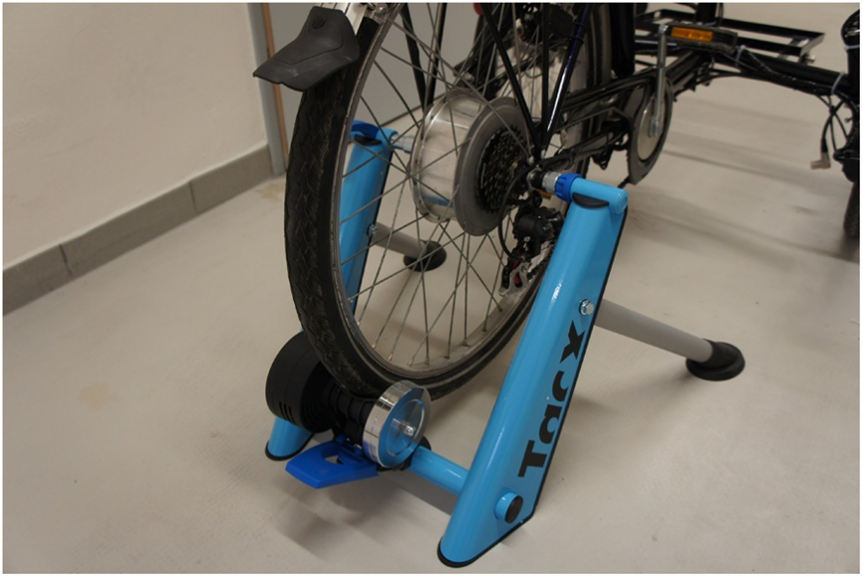
**E-bike mounted on a bicycle trainer.**

The measurements were carried out in a healthy subject and for the measurements, the bike was mounted to a bicycle trainer (see Figure 4).

The function of the ultrasonic sensors was tested. Depending on the distance the frequency of the acoustic signal changes (the lower the distance, the higher the frequency). With the support of a measuring tape we could prove that the distance measured by the ultrasonic sensors was correct. Narrow passages or objects that were nearby the bicycle were registered from the different placed sensor. The position of the bicycle relative to the object has been signaled acoustically depending on the altered distance. The acoustic signal was loud enough so that the biker was able to recognize it even in noisy traffic conditions. We also proved that the blinking light was placed well so that other road users could get information about the size of the bike.

In the first experiment, the cycling was done without the use of the electromotor. In the second experiment, the electromotor was involved. Before and after cycling, the blood pressure of the test person was measured. The blood oxygen saturation and the heart frequency were measured continuously during cycling.

To make the measurement results comparable, the velocities in both cycling experiments should be equal. The velocities and the pedaling rate were recorded during the whole cycling tests. Also the pedaling force was continuously measured and could be afterwards evaluated with a LabVIEW-programme.

The measured values of the blood pressure are presented in Table 1. Compared to cycling with electromotor, there was a sharp increase in the blood pressure after cycling without electromotor.

**Figure 5:**
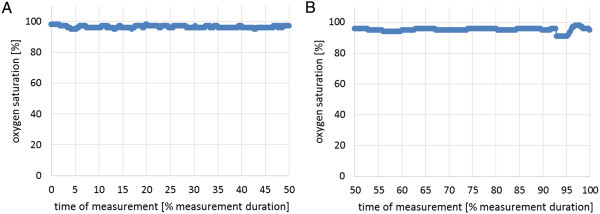
**Blood oxygen saturation in cycling experiment. (A)** without electromotor and **(B)** with electromotor.

The results of the blood oxygen saturation are shown in Figure 5A (without electromotor) and 5B (with electromotor). No essential differences were registered between cycling with and without electromotor.

**Figure 6:**
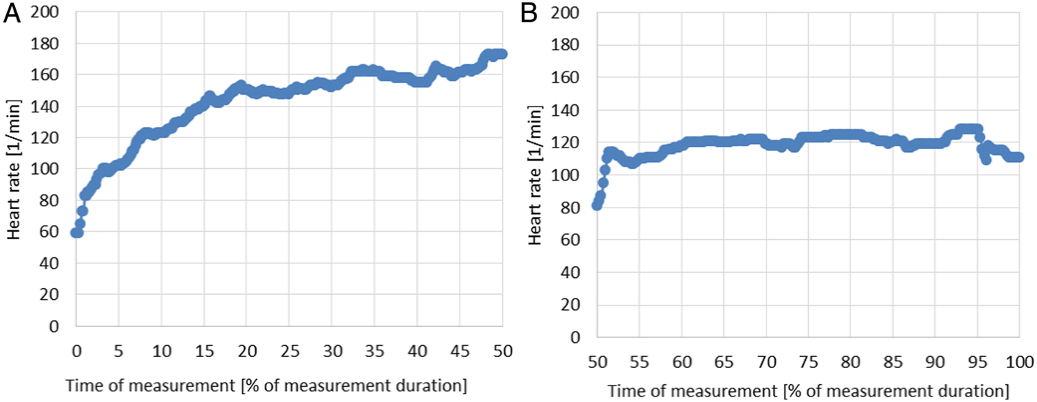
**Heart rate during cycling. (A)** without electromotor; **(B)** with electromotor.

The heart rate course during cycling is shown in Figure 6A (without electromotor) and 6B (with electromotor). Already after 3 minutes cycling without electromotor, the heart rate has reached the value 160 beats per minute and is slowly increasing until the end of the cycle. After turning-on the motor, the heart rate did not exceed 120 beats per minute during the whole measurement time, which means that the motor supported the biker and helped not to overstrain him.

**Figure 7:**
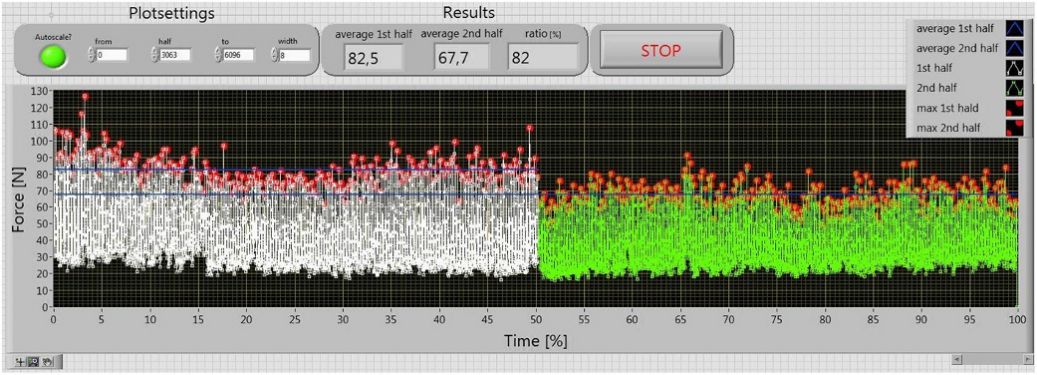
**Measurement of the pedaling force over the whole measurement time.**

**Figure 8:**
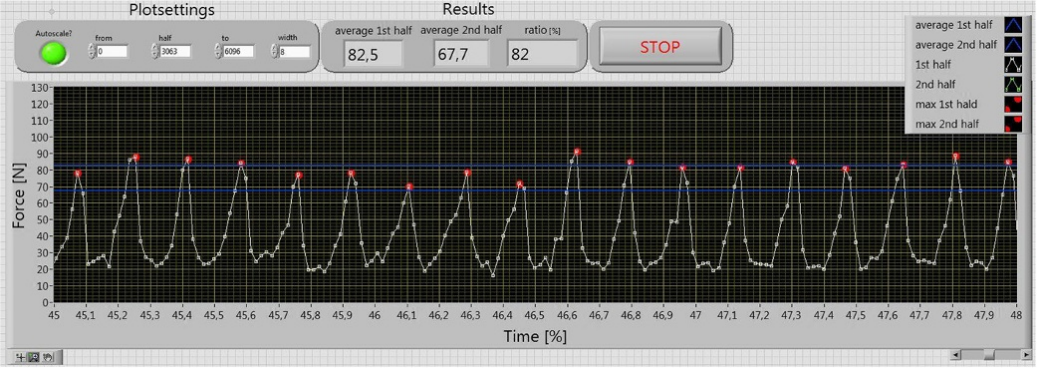
**Measurement of the pedaling force in detail.**

Figures 7 and 8 demonstrate the evaluation of the force measurement. In the evaluation, the maximal values (marked with red points) were split in two ranges, averaged, and compared. The approximate maximum value in the measurement with electromotor was 18% lower than the one in the measurement without electromotor. After the half of the time, the average maximum value of the force dropped down to less than 70 N. This means that the force which is needed to hold the constant velocity decreases.

**Table 1:** **Blood pressure values before and after biking**

Time of measurement	Before 1st measuring cycle	After 1st measuring cycle	Before 2nd measuring cycle	After 2nd measuring cycle
Blood pressure	117/76	194/105	112/81	120/83

## Discussion

People with cerebral palsy are limited in their ability to walk and severely restricted because of their motoric impairment. Nevertheless, Maggs et al. showed in ( 2011) that young people with cerebral palsy give their priorities for physical activity. According to Rimmer and Rowland ( 2008), increase of physical activity and fitness among youth with disabilities is one of the most important challenges for pediatric rehabilitation. Wheelchairs, bicycles and various options help to cover longer distances and have an additional therapeutic effect. Through regular bike training, coordination, balance, but also physical condition can be improved effectively and facilitate to prevent contractures.

Besides the motor disabilities, perceptive deficits should be kept in mind when cycling. Bikes for people with disabilities have to offer adequate stability which is provided by a tricycle configuration. Also adjustments, such as additional foot straps to fix the feet on the pedals are common. Moreover, E-bikes increasingly enter the market and trikes are also successfully adapted with electric motors.

This work describes how a commercial trike with already integrated E-bike motor, was additionally equipped with self-developed devices which helps to compensate the perception problems. For this purpose, an ultrasonic distance sensors system was developed and adapted. It works similar to a parking sensor in a car. If a defined threshold distance of the bicycle to another object is registered, an acoustic feedback is generated. Also, a force plate was developed which enables testing to what extent the E-bike motor provides a reduction in the use of muscle power. To confirm that the E-bike motor helps not to overstrain the patient, measurements of health parameters like heart rate and blood pressure were done with and without the use of the motor.

To provide accurate and uninterrupted data records, the heart rate, blood pressure and pedaling force measurements were done while mounting the bike on a bicycle trainer. This method with a stationary bike was also used by Bar-Haim et al. ( 2007) to ensure an accurate measurement. The function of the ultrasonic measuring system for evaluating the distances to objects was tested during cruising. It was confirmed that the bicycle was able to detect obstacles and to assess distances to objects.

The well-functioning distance measurement system gives hope that it can be helpful to compensate deficits in perception and provide support in stressful situations, such as biking in the traffic. The audio feedback of the measurement system is well perceptible in noisy environments. For persons with acoustic impairments, the distance warning system could also be supplemented by optical options, so that the distances to obstacles could be signalized by, for example, a colored light warning system.

## Conclusions

An adapted E-bike can be improved to provide better space orientation for people with perceptual disorders and to measure training conditions of patients. Moreover, these enable individual adjustment of the electric motor power to optimize comfort and therapy effect.
